# Office workers’ beliefs about reducing sitting time at work: a belief elicitation study

**DOI:** 10.1080/21642850.2018.1428103

**Published:** 2018-01-29

**Authors:** Ailsa Niven, Dan Hu

**Affiliations:** Physical Activity and Health Research Centre, Institute of Sport PE and Health Sciences, University of Edinburgh, Edinburgh, UK

**Keywords:** Sedentary, theory of planned behaviour, sitting behaviour

## Abstract

**Objectives:**

Prolonged sitting has adverse health consequences, yet office workers can spend over 10 hours sitting each day. The Theory of Planned Behaviour may offer a useful perspective for understanding and enhancing psychological determinants of sitting at work. The aim of this belief elicitation study was to identify office workers’ most salient beliefs relating to achieving the recently published Public Health England recommendation of accumulating at least two hours per day of standing and light activity at work.

**Methods:**

Full-time office-based workers (*n* = 105) responded to our invitation on Twitter to complete an on-line questionnaire. Participants responded to six open-ended questions about their behavioural (i.e. advantages/disadvantages), normative (i.e. who would approve/disapprove), and control (i.e. easy/difficult) beliefs relating to the target behaviour, and the data were content analysed to identify the most salient themes.

**Results:**

The most salient advantage of the behaviour was better health (*n* = 243), and most salient disadvantage was decreased work productivity (*n* = 64). Participants believed that people in work with a remit for health (*n* = 34) were likely to approve of the behaviour, but that managers (*n* = 68) would be likely to disapprove. It was believed that a better physical environment (*n* = 75) would make it easier, and work demands (*n* = 102) would make it difficult to execute the behaviour.

**Conclusions:**

Although participants recognised many benefits of engaging in the behaviour, there was consistent evidence that participants believed the behaviour may have implications for working effectively, and would be influenced by the physical environment and work culture. Interventions should target these salient beliefs.

## Introduction

Sedentary behaviour is defined as any waking behaviour characterised by an energy expenditure of ≤1.5 Metabolic Equivalents, whilst in a sitting or reclining posture (Sedentary Behaviour Research, 2012; Tremblay et al., 2017). Considerable evidence now indicates that sedentary behaviour is strongly associated with an increased risk of cardiovascular disease, type 2 diabetes, metabolic syndrome, some cancers and all-cause mortality (de Rezende, Lopes, Rey-Lopez, Matsudo, & Luiz, 2014; van der Berg et al., 2016). Nevertheless, many adults spend a large proportion of their day being sedentary (Bennie et al., 2013). Office workers can spend over 10 hours sitting each day, and have been identified as at particular risk of the health consequences of prolonged sedentary behaviour (Smith et al., 2015). In 2015, an expert statement commissioned by Public Health England and the Active Working Community Interest Group was published to provide guidance on reducing sedentary behaviour at work. The expert statement recommended that predominantly desk-based workers should aim to ‘initially progress towards accumulating at least 2 h/day of standing and light activity (light walking) during working hours’, and regularly break up sitting behaviour (Buckley et al., 2015, p. 2). However, there is some evidence that these recommendations have been met with initial scepticism by the public (Gardner, Smith, & Mansfield, 2017).

There has been a growth in intervention studies aiming to reduce sitting time in the office workplace using a number of strategies including environmental (e.g. height adjustable desks), educational/behavioural (e.g. goal setting), and multi-component interventions (Chu et al., 2016). From their systematic review, Chu et al. (2016) concluded that multi-component interventions were likely to be most effective, with a reduction of daily workplace sitting of 1.5 hours. Despite the promise of these interventions, there is still limited understanding of the factors that influence sitting behaviour at work (Prapavessis, Gaston, & DeJesus, 2015). Extending understanding of the determinants of sitting at work will further enhance intervention design and effectiveness (Michie, van Stralen, & West, 2011).

It is likely that the determinants of sitting behaviour at work will be multi-factorial and interactive reflecting broad social-ecological (Owen et al., 2011), and more recent system-based perspectives (Chastin et al., 2016). Psychological factors are identified within each of these perspectives. However, there has been limited research drawing on psychological theories of behaviour change to understand and influence sedentary behaviour, despite the potential modifiable nature of these determinants that may be targeted in interventions. Recently, Rollo, Gaston, and Prapavessis (2016) undertook a systematic review of cognitive and motivational factors associated with sedentary behaviour. The findings based on 25 identified studies highlighted a number of factors associated with sedentarism including intentions, attitude, motivation, social support/norms, self-efficacy/control beliefs, and habit. It was notable that only six of the reviewed studies were grounded in social-cognitive and motivational models, such as the Theory of Planned Behaviour (Ajzen, 1991) highlighting the dearth of theoretical research in this area. Although the review makes an important and timely contribution to the field, the review included different groups (i.e. adults, children, adolescent), and different types of sedentary behaviour (i.e. total and domain-specific behaviours such as TV viewing) that are likely to moderate the relationship between these psychological variables and sedentary behaviour.

Addressing the need for further theoretical research that addresses different domains of sedentary behaviour, Prapavessis et al. (2015) examined the utility of TPB to predict adult (*n* = 372) sedentary behaviour generally, and in different domains including weekday and weekend, and volitional (i.e. leisure) and non-volitional (i.e. work/school) behaviour. The findings showed that attitude, perceived behavioural control, and subjective norm explained between 9% and 58% of the variance in intention, with a stronger relationship with non-volitional tasks such as work/school, compared with volitional tasks such as TV viewing. Together these constructs predicted between 8% and 43% of the variance in sedentary behaviour, with predictive superiority for the behaviour of weekday work/school. These findings provide preliminary evidence that suggests the TPB could offer a useful framework for understanding sedentary intention and behaviour, particularly for work/school related behaviours.

However, there are two limitations of Prapavessis et al. (2015) study that require further consideration. Firstly, the target behaviour was current sedentary behaviour not reduction in sedentary behaviour. It is probable that there will be different cognitive and motivational determinants relating to the adoption of the new health behaviour of reducing sedentary time through increased standing and light activity, compared with current sedentary behaviour (Schwarzer, 2008). For example, attitudes towards the existing behaviour of sitting will likely differ, and could have a different relationship with the new behaviour of reducing sitting time. In order to develop interventions to change sedentary behaviour, it would be of particular value to consider how the TPB can contribute to understanding the adoption of the new behaviour rather than the continuation of an existing behaviour.

Secondly, Prapavessis et al. (2015) did not undertake an elicitation study. Although not always undertaken, it has been recommended that when applying the TPB to a new behaviour or population, researchers should undertake a belief elicitation study in order to determine the salient behavioural, normative, and control beliefs of the population (Ajzen & Fishbein, 1980; Fishbein & Ajzen, 2010). These beliefs are viewed as the cognitive foundation of behaviour, which operate through the TPB constructs and can provide substantive information to help explain (not just predict) behaviour (Fishbein & Ajzen). Behavioural beliefs influence individuals’ attitudes, and are individuals’ views on the consequences of engaging in the target behaviour (e.g. disrupt work). Normative beliefs relate to the subjective norm construct, and relate to individuals’ views as to whether significant others approve or disapprove of the behaviour (e.g. manager). Control beliefs relate to perceived behavioural control, and reflect individuals’ beliefs about what would make it easier or more difficult to engage in a new behaviour (e.g. office environment). Belief elicitation studies are undertaken to identify which modal salient beliefs most strongly influence attitudes, subjective norm and perceived behavioural control in a specific group. A comprehensive evaluation of the utility of the TPB examines the full pathway from beliefs to behaviour; however, there is limited use of elicitation studies in some areas, such as exercise TPB research (Downs & Hausenblas, 2005). In addition to contributing to TPB research, a belief elicitation study can have merit as a standalone study by providing valuable information on specific groups’ thoughts and feelings about a target behaviour (Bellows-Riecken, Mark, & Rhodes, 2013; Darker, French, Longdon, Morris, & Eves, 2007; Downs & Hausenblas, 2005). This information can be used to inform interventions as argued by Fishbein and Ajzen (2010):

By identifying the behavioral, normative, and control beliefs that serve as the underlying determinants of a behavior we also gain important information about the kinds of beliefs that would have to be changed to effect a change in intentions and behavior. (p. 322)

For example, identifying salient behavioural beliefs could then inform the content of health promotion messages to promote attitudinal change towards reducing sitting behaviour at work. Whilst previous research has considered office workers’ views on reducing sedentary time at work (e.g. Cole, Tully, & Cupples, 2015; Hadgraft et al., 2016), this has been from a non-theoretical perspective.

To summarise, office workers spend large proportions of their day being sedentary despite accumulating evidence demonstrating the health risk of this behaviour. The effectiveness of interventions may be enhanced through greater understanding of the determinants of the sedentary behaviour at work, although limited research has drawn on established psychological theories of behaviour change. There is increasing interest in using the TPB to understand sedentary behaviour; however, to the best of our knowledge, no belief elicitation study has been undertaken to identify the cognitive foundations of reducing sitting behaviour at work that can be used to inform interventions. The aim of this study was to identify office workers’ most salient beliefs relating to achieving the Public Health England recommendation of accumulating at least 2 hours per day of standing and light activity at work. The study focused on the Public Health England recommendations because these are likely to inform health promotion initiatives targeting U.K. office-based workers.

## Method

### Research design

This was a qualitative study based on Fishbein and Ajzen (2010) recommendations for undertaking a belief elicitation study.

### Participants

Participants were full-time office-based workers (*n* = 105; male = 26) from a range of age groups (18–24 = 7.3%; 25–34 = 24.8%; 35–44 = 28.4%; 45–54 = 24.8%; 55+ = 14.7%) and different employment sectors (public sector = 61%; private sector = 12.4%; charity = 26%; other = 1%) in the United Kingdom. Based on the Occupational Sitting and Physical Activity Questionnaire (OSPAQ) (Chau, Van der Ploeg, Dunn, Kurko, & Bauman, 2012), participants self-reported working on average 36.49 (SD = 5.93) hours per week, and spending 357 (SD = 97) minutes sitting, 37 (SD = 54) minutes standing, 56 (SD = 46) minutes walking, and 3 (SD = 12) minutes doing heavy labour or physically demanding tasks on a typical work day.

### Instrument

Based on the recommendations of Fishbein and Ajzen (2010) for undertaking a belief elicitation study, six open-ended questions were created to identify beliefs towards the target behaviour of accumulating at least 2 hours per day of standing and light activity (e.g. walking) at work. The target behaviour was repeatedly presented in each question for emphasis. Two items related to behavioural beliefs: ‘what do you see as the advantages of accumulating … ’ and ‘what do you see as the disadvantages of accumulating … ’. Questions related to normative beliefs included two items targeting injunctive norms: ‘Please list the individuals or groups who would approve or think you should … ?’ and ‘Please list the individuals or groups who would disapprove or think you should not  … ?’ Finally, participants were asked two items in order to address control beliefs: ‘Please list any factors or circumstances that would make it easy or enable you to … ’ and ‘Please list any factors or circumstances that would make it difficult or prevent you from … ’. An answer to each question was required and there was no limit on the number of characters that could be entered. The questionnaire was piloted with participants (*n* = 2) representative of the sample, and no changes were made.

### Procedure

Ethical approval was granted by Moray House School of Education, University of Edinburgh ethics committee. Individuals were recruited between June and July 2016, primarily through responding to a tweet on Twitter inviting full-time office-based workers to complete an on-line survey on sitting at work. The tweet tagged nine national health-related institutions to encourage retweeting to extend the potential audience. Additionally, a health-promoting organisation disseminated the questionnaire link via their e-news. The questionnaire link directed participants to information about the study, and in order to start the questionnaire participants were required to confirm their consent to participate using a tick box. As an incentive to participate, participants were given the option to enter a prize draw for one of two £20 vouchers by entering their email address at the end of the questionnaire. At the end of the one-month data collection period, data were downloaded from the on-line survey into excel files.

### Data analysis

Descriptive data from the OSPAQ were calculated by multiplying the percentage of time in each activity by the number of self-reported hours worked per day to provide an indication of the sitting and physical activity behaviour at work (Chau et al., 2012). Consistent with similar research (Bellows-Riecken et al., 2013) and consistent with the recommendations of Fishbein and Ajzen (2010), the free-response data were content analysed to identify themes within each of the beliefs. Two researchers worked together to identify major themes by following four steps that incorporated: (i) coding the raw data to provide an adequate description of the response; (ii) clustering codes with similar content into sub-themes; (iii) assessing sub-themes for internal coherence and to determine if they could be further clustered into higher order themes; (iv) if further themes could be developed, the third step was repeated with these themes. A frequency count of the number of codes within each theme was recorded in order to identify the most salient beliefs.

## Results

Tables 1–3 summarises the themes that were identified, the frequency count for each theme, and an example quote in relation to the behavioural, normative, and control beliefs, respectively.

**Table 1.** Emergent themes, frequency count and example quotes relating to behavioural beliefs about accumulating 2 hours per day of standing and light activity during working hours.

Belief type/theme	Frequency	Example quote
Advantages Better health		
Muculoskeletal	104	‘better for posture, rather than slouching at desk, and its impact on the back’
Physical	42	‘a break away from computer screen to prevent strain on eyes and sore heads’
Mental	33	‘Clear(s) your head and stops you from thinking about work’
General	28	‘good for health’
Reducing fatigue	33	‘prevents tiredness’
Enhance digestion	3	‘better digestion’
Enhance work efficiency	47	‘helps with concentration when returning to desk’
Social benefits	15	‘Adds a social side to the day – that is, meeting/greeting colleagues in the corridor etc.’
Have a break	19	‘gives you a break from looking at a computer screen’
Disadvantages		
Decreased work productivity		
Cannot work	21	‘two hours standing/walking out of a seven hour day is quite a lot of time not to be working’
Less productive	21	‘not being as productive’
Disrupt work	7	‘Disruption to getting work done’
Break concentration	4	‘Break in concentration of task’
Time consuming	11	‘Less time to finish work’
Concern what others think	14	‘Other people perceive lack of commitment to work if not at desk’
Negative health conseq	12	‘back pain’
Job not conducive	12	‘ … my work revolves around internet databases – not so easy to access while active’
Negative conseq for others	6	‘other people have to answer telephone etc. on your behalf’

**Table 2.** Emergent themes, frequency count and example quotes relating to normative beliefs about accumulating 2 hours per day of standing and light activity during working hours.

Belief type/theme	Frequency	Example quote
Approve		* *
Everyone	7	‘everyone should approve of this as it is essential to the wellbeing of employees’
Myself	3	‘Me!’
None	7	‘None – feel I am alone in wishing I could move more’
People within work		
Those with remit for health	34	‘Those involved in the health and welfare of staff’
Managers	31	‘Line managers’
Colleagues	34	‘Colleagues’
Those who sit a lot	6	‘Colleagues who sit all day’
Out with work		
Health professionals	22	‘Health professionals’
External agencies	6	‘Healthy working lives staff’
Friends and family	4	‘Only friends’
Health conscious	4	‘Healthy active people interested in their own wellbeing’
Disapprove		
No one	18	‘No one I can think of’
Unsure	8	‘Not sure’
Managers	68	‘Managers – perhaps might think you're walking and therefore not working’
Colleagues	18	‘colleagues I share a desk’
Overworked colleagues	5	‘other colleagues who have more work than you or who are tied to their desks or lab work more’
Those unable to	10	‘Anyone not able to stand’
Unhealthy lifestyle	4	‘People who don’t exercise and have unhealthy lifestyles’

**Table 3.** Emergent themes, frequency count and example quotes relating to controls beliefs about accumulating 2 hours per day of standing and light activity during working hours.

Belief type/theme	Frequency	Example quote
Easier		
None	1	‘none’
Physical work environment		‘a break away from computer screen to prevent strain on eyes and sore heads’
Standing desks	33	‘A chance to get my desk set up to enable me to stand’
Adjustable desks	3	‘adjustable desks’
Treadmill desks	3	‘treadmill desks’
No access to lift	2	‘not having/choosing not to use a lift and taking the stairs’
Non-open plan office	2	‘if we didn't work in an open plan office where all contacts were around you … ’
Designated areas	3	‘Break out areas for ad hoc meetings/ catch-ups’
Greater distance to complete task	7	‘having tea and coffee making facilities further away from the desk space’
More attractive outside area	3	‘Having somewhere nice to walk outside’
Flexible space/technology	9	‘use of tablets rather than PCs’
Add objects/reminders	6	‘something in your calendar to remind you to get up every so often to stretch your legs’
Work culture		
Acceptance of behaviour	34	‘an agreement from management that people are expected to take short breaks (rather than the exact opposite where people are challenged for time spent away from the desk)’
Walking/standing meetings	23	‘Walking meetings or quick standing meetings’
Work demands	8	‘lighter workload’
Flexibility	7	‘being allowed to manage my own time’
Job type	6	‘A more active role. My role is very desk based’
Having regular breaks	6	‘Giving me a two hour lunch break in the middle of the day?’
Workplace walking initiatives	9	‘Walking groups at lunch time’
Self-motivation	2	‘Being strong yourself and just getting up and walking away’
Difficult		
None	2	
Not sure	2	
Work demands		
Too much work	47	‘Time constraints / increasing workloads and pressures’
Too many meetings	15	‘number of meetings to attend on weekly basis’
Nature of the job	40	‘The constant barrage of emails that have to be dealt with’
Physical environment		
Lack of equipment	14	‘can’t work standing or walking without the equipment for this’
Nature of the building	8	‘kitchen and toilet close to office’
Lack of outdoor space	5	‘if there was not a lot of walking space near by’
Poor weather	8	‘Raining outside or bad weather’
Work culture		
Colleague disapproval	14	‘who is in the office that might disapprove’
Lack of cultural acceptance	13	‘Work place culture that does not recognise benefits’
Habit	1	‘the only thing preventing me spending 2 hours standing or walking during work is the habit of not doing it’
Health	1	‘health’
Not able to identify ways to sit less	1	‘ways of thinking on how to vary the work from day to day’

### Behavioural beliefs: what are the advantages and disadvantages of the behaviour

In total 324 perceived advantages of accumulating at least 2 hours per day of standing and light activity (e.g. walking) at work were reported, with two participants identifying no benefits. The largest theme was labelled better health (*n* = 243) and was made up of sub-themes relating to improved musculoskeletal health (*n* = 104), better physical (*n* = 42), mental (*n* = 33), and general (*n* = 28) health, as well as reducing fatigue (*n* = 33), and enhancing digestion (*n* = 3). The most salient theme of musculoskeletal health included a number of comments such as reducing back pain and enhancing mobility. For example, one participant stated that the target behaviour is ‘better for posture, rather than slouching at desk, and its impact on the back’. Comments relating to physical health were varied relating to fitness, weight management, and as illustrated by one participant providing: ‘a break away from computer screen to prevent strain on eyes and sore heads’. Comments relating to mental health were also varied and included perceptions of enhanced well-being, more positive feelings, less boredom, and, as illustrated by one participant standing and moving around helps ‘Clear(s) your head and stops you from thinking about work.’

The second most salient theme related to participants identifying that standing and moving could enhance work efficiency (*n* = 47), because they could work more productively, and concentrate and think better. For example, one participant suggested that it ‘helps with concentration when returning to desk’. Less salient themes identified by participants were that standing and moving more would have social benefits (*n* = 15) by encouraging greater interaction with colleagues in the workplace. Participants also identified having a break (*n* = 19) as an advantage to have a break in work and in sitting specifically, and also having an opportunity to leave the office.

In relation to perceived disadvantages of accumulating 2 hours of standing and/or light activity, 108 comments were reported, with 20 participants reporting no disadvantages. The most salient theme related to the perception that this behaviour would decrease work productivity (*n* = 64). The theme decreased work productivity reflected concerns that individuals cannot work at the same time as standing (*n* = 21), as illustrated by one participant who reported ‘two hours standing/walking out of a seven hour day is quite a lot of time not to be working’*.* Further participants perceived that they would be less productive (*n* = 21), the behaviour would disrupt work (*n* = 7), break concentration (*n* = 4), and be time consuming (*n* = 11). Further perceived disadvantages related to being concerned about what others would think about them engaging in the behaviour (*n* = 14), that there would be negative health consequences (*n* = 12), that the job demands or physical environment were not conducive, and finally that the behaviour may have negative consequences for others at work (*n* = 6).

### Normative beliefs: who would approve and disapprove of the behaviour?

Participants made 158 comments relating to who would approve of the behaviour, with some participants identifying ‘everyone’ (*n* = 7), ‘myself’ (*n* = 3), and ‘no one’ (*n* = 7), and additionally two participants responded that they were uncertain who would approve. The remaining comments were organised into themes relating to people at work (*n* = 105), and people out with work (*n* = 36). In the work environment, it was perceived that the behaviour would be approved of by those with a remit for health at work (*n* = 34) such as health safety, occupational health, human resources, and employees related to health promotion. Participants also reported that managers (*n* = 31), colleagues (*n* = 34), and those who sit a lot (*n* = 6) would approve of the behaviour. Out with work, participants identified that health professionals (*n* = 22), and external agencies (*n* = 6) with a role for promoting activity and health would approve. Additionally, limited numbers of participants reported that friends and family (*n* = 4), and health conscious individuals (*n* = 4) would approve.

In terms of who would disapprove of the behaviour, participants made 131 comments. Some participants identified that no one would disapprove (*n* = 18), and eight were unsure. The majority of the comments (*n*= 91) related to people in work and specifically managers (*n* = 68), colleagues (*n* = 18), and overworked colleagues (*n* = 5). For example, one participant reported those who would disapprove would be ‘people that think you are just skiving – maybe managers or people from other teams’. Similarly, another participant reported ‘Managers – perhaps might think you're walking and therefore not working’ highlighting the perception that managers would disapprove because it would be perceived that employees are not working if they are standing or moving around. Participants also indicated that those who were unable to do the behaviour (*n* = 10) because they were inactive, unable, have a disability, or elderly, and those with an unhealthy lifestyle (*n* = 4) would also disapprove.

### Control beliefs: what factors would make it easy or difficult to engage in the behaviour?

Participants identified 169 factors that would make it easier for them to engage in the behaviour, and additionally two participants indicated that they were ‘not sure’ and one participant replied ‘none’. The two largest themes related to clusters of comments about changes in the physical work environment (*n* = 75), and changes to the work culture (*n* = 84). In relation to the physical environment, a number of comments were made about how use of standing (*n* = 33), adjustable (*n* = 3), and treadmill (*n* = 3) desks would facilitate standing. It was also suggested that changing the physical layout of the work environment would be helpful (*n* = 21), so that there was no access to the lift (*n* = 2), the office was not open plan (*n* = 2), and bigger (*n* = 4), employees had a designated area to have a stand/move (*n* = 3), and that employees had to move more to undertake tasks (*n* = 7). For example, participants suggested that ‘having tea and coffee making facilities further away from the desk space’ or ‘having to take mail to a centralised collection point’ would make it easier to do the behaviour. A small number of participants indicated that if the environment outside the office was more attractive (*n* = 3) then this would encourage them to walk more. In addition to suggested changes to the infrastructure, participants also commented that creating flexible working space with the use of moveable technology (*n *= 9), such as mobile phones and tablets, would make it easier for them to stand and move around. Finally, some participants suggested that adding objects to the environment such as a monitoring device like a pedometer (*n* = 2), and a reminder (*n* = 4) to break up sitting such as putting ‘something in your calendar to remind you to get up every so often to stretch your legs’ would help.

Participants also commented that changing their work culture and how they work may facilitate engaging in the behaviour. Specifically, a number of comments related to how an increased acceptance of the behaviour and engagement in the behaviour, particularly from managers, would help create a culture change (*n* = 34) that would make it easier to break up sitting. For example, one participant commented that it would be easier if there was ‘an agreement from management that people are expected to take short breaks (rather than the exact opposite where people are challenged for time spent away from the desk)’. A number of participants made comments (*n* = 23) that increasing the number of walking and standing meetings, and meetings outside the office would also provide an opportunity to move more. Participants also noted that if the nature of their job (*n* = 21) in terms of the work demands (*n* = 8), flexibility (*n* = 7) in ‘being allowed to manage my own time’, and job type (*n* = 6) were changed then it would help. Having regular breaks and dedicated time (*n* = 6) to break up sitting were also identified as factors that could encourage the behaviour. Finally, out with these two large themes, it was suggested that engaging workplace walking initiatives (*n* = 9) and being self-motivated (*n* = 2) would make it easier to complete the behaviour.

Participants identified 167 factors or circumstances that would make it difficult for them or prevent them from accumulating at least 2 hours per day of standing and light activity at work. Additionally, two participants suggested there were no factors, and two participants were unsure of any factors. The largest theme was labelled Work Demands (*n* = 102) and included comments by participants about having too much work to do (*n* = 47) and having too many meetings (*n* = 15). For example, participants highlighted ‘intense workload’, ‘heavy workload’, and ‘too much work’ as barriers. Participants also indicated how the nature of their jobs (*n* = 40), which were desk and computer-based, required responding to emails, or incorporated travel by train or car, would stop them achieving the behaviour. For example, one participant stated ‘It's hard to be able to get away when your work is 100% PC based’. The second largest theme related to the physical work environment (*n* = 35) and how aspects of the workplace prevented them from achieving the behaviour. Lack of equipment (*n* = 14), and specifically standing desks and flexible technology were barriers as highlighted by one participant ‘Can't work standing or walking without equipment for this’. The nature of the office building (*n* = 8) being small, open-planned or with no changing facilities, lack of outside space (*n* = 5), and the poor weather (*n* = 8) were also identified as barriers. The work culture emerged as a third theme and related to managerial or colleague disapproval for the behaviour (*n* = 14), and a lack of cultural acceptance for the behaviour (*n* = 13). For example, one participant stated their work place had a ‘culture that does not recognise benefits’ of reduced sitting. Finally, a small number of participants suggested that individual-level barriers to changing behaviour including siting as a habit (*n* = 1), health (*n* = 1), and not being able to identify ways to sit less (*n* = 1).

## Discussion

Beliefs that office workers have about reducing their sitting time are the underlying cognitive determinants of the behaviour (Fishbein & Ajzen, 2010). To the best of our knowledge, this belief elicitation study is the first undertaken in the area of sedentary behaviour and contributes to the growing theoretical literature by providing insight into the salient behavioural, normative and control beliefs sedentary U.K. office workers have towards achieving the Public Health England recommendation of accumulating at least 2 hours per day of standing and light activity at work. This discussion will focus on the most salient themes to consider strategies to target those beliefs that are more likely to influence determinants of, and behaviour in this group (Fishbein & Ajzen, 2010).

Behavioural beliefs underpin individuals’ attitude towards a behaviour, and both advantages and disadvantages to engaging in the target behaviour were identified. Encouragingly participants identified double the number of advantages, and 20 participants did not identify any disadvantages. The most salient perceived advantages related to the physical and mental health benefits that would be obtained, and particularly to musculoskeletal health to improve posture and reduce pain. Whilst there is a growing body of literature demonstrating the chronic consequences of sedentary behaviour on some aspects of health (e.g. all-cause mortality, cardiovascular disease), there is inconclusive evidence of the musculoskeletal benefits (de Rezende et al., 2014; Neuhaus et al., 2014). Nevertheless, two recent studies highlighted the benefits of environmental interventions designed to reduce sitting for subjectively assessed lower back pain (Foley, Engelen, Gale, Bauman, & Mackey, 2016), and neck and shoulder discomfort (Gao, Nevala, Cronin, & Finni, 2016). However, there is a need for further research to provide more robust evidence to support office worker’s anecdotal beliefs about the acute and chronic musculoskeletal benefits of reduced sitting. It is possible that the beliefs about musculoskeletal benefits are more salient and important because they are experienced more immediately following breaking up of sitting, compared with longer-term benefits to other chronic conditions. Promoting and reinforcing these immediate well-being benefits from enacting the behaviour may help to promote behaviour change. Health messaging may offer a cost-effective and wide-reaching intervention strategy to promote these positive beliefs in order to enhance determinants of and actual sitting behaviour. These salient positive beliefs could form the basis for persuasive communication interventions designed to target office workers through gain-framed messages (Rothman & Salovey, 1997). Although there is a body of research examining messaging in other health behaviours (Gallagher & Updegraff, 2012), limited research has considered the value of messaging for promoting reduced sitting.

The second most salient advantage theme related to how the behaviour could lead to enhanced work efficiency, as a break could lead to enhanced concentration and thinking. However contrary to this, decreased work productivity was the most salient theme related to disadvantages, and highlights the mixed views. Many participants reported that it would be difficult to be productive whilst standing or moving, and that engaging in the behaviour would disrupt their work and concentration. This perception of being unable to work effectively whilst reducing sitting is consistent with other qualitative findings (Cole et al., 2015), and is clearly a belief that would need to be challenged to facilitate behaviour change. Indeed, there is some evidence that the introduction of activity-permissive workstation desks does not change work productivity (Chau et al., 2014; Hadgraft et al., 2017; Neuhaus et al., 2014). Interventions could provide guidance on or opportunities to experience how to work efficiently whilst reducing sitting in order to target this belief. For example, guidance on conducting effective walking meetings, or highlighting tasks that can be undertaken standing up may be useful.

According to the TPB, beliefs about whether significant others will approve or disapprove of the target behaviour will influence intention and behaviour. The most salient theme relating to those who would approve of the behaviour related to those in the workplace, and included those who had a remit for health within the workplace, and colleagues and managers. This finding is encouraging suggesting that the workplace may be supportive of reduced sitting. However, it was evident that participants also believed that their work colleagues, and especially managers, would be the main group who would disapprove of the behaviour. Some comments indicated that participants believed that if they were not sitting then they would be perceived to be not working. This belief links with the disadvantages noted above that the target behaviour is incompatible with working effectively. Previous research has also highlighted how managers may disapprove of reduced sitting because it may influence productivity (Cole et al., 2015), and these findings highlight the importance of engaging management endorsement (and involvement) as part of any intervention (Dunstan et al., 2013).

Participants identified a number of, predominantly external, factors that would make it easier or more difficult for them to engage in the behaviour. Changes to both the physical work environment and the work culture were the most salient themes relating to facilitating undertaking the behaviour. Many participants were aware of standing/adjustable desks and highlighted that their introduction would be helpful, although this may be prohibitively costly. Participants believed that a shift in culture to increase the acceptability, wider participation, and management endorsement of increased standing and moving was required to facilitate change. This finding is consistent with previous research that suggests workers perceive managers/employers as the key gatekeepers to the implementation of the recommendations (Gardner et al., 2017), and further supports the need for engaging management in interventions. Consistent with Hadgraft et al. (2016), participants also suggested that work practices could change to include more standing and walking meetings. In terms of what made it difficult for participants to reduce sitting, the largest theme clustered comments relating to the heavy demands of the work, and the nature of the role that required them to be desk- and computer-based. This finding highlights, once again, the belief that reducing sitting is incompatible with working effectively and the need to challenge this. Overall, participants primarily identified external factors that would influence their ability to engage in the behaviour, and it is notable that few individual factors such as confidence, self-regulation, and habit were identified. Interventions targeting both the workplace physical environment and culture would be required to enhance individuals’ beliefs that they can engage in the behaviour, and is consistent with evidence suggesting the superior effectiveness of multi-component interventions (Chu et al., 2016).

A strength of this study is that it uses a theoretical perspective to increase understanding of office-based workers’ beliefs relating to the Public Health England recommendation of accumulating 2 hours of standing or light activity during the working day. Drawing on theory provides direction for future interventions by identifying key constructs to target, and also provides insight into why or why not an intervention was effective in changing behaviour (Michie & Prestwich, 2010). Focusing the study on the specific behaviour recommended by Public Health England is also a strength, because this recommendation is likely to be the basis for health promotion strategies and the findings will therefore have relevance and implications for these initiatives. Using Twitter to recruit participants can be viewed as both a strength and a shortcoming of the study. Using Twitter to recruit research participants is relatively novel, and has the potential to efficiently reach a wide number of participants from a range of workplaces. However, it is difficult to determine how representative the sample is of the potential population. In this study, a sample was recruited that was largely representative of a range of work sectors and age groups, but had a gender imbalance. Further, it is acknowledged, that the sample may have been biased because health-related organisations re-tweeted the message, and their followers may already have an interest in health. Nevertheless, the findings were not strongly positive toward reducing sitting suggesting limited bias. Future research could sample other groups in order to consider the generalisability of the findings, and also the potential moderating role of factors such as age, gender and job type.

To conclude, although participants recognised many benefits of engaging in the behaviour of accumulating 2 hours of standing or light activity during the working day, overall there was consistent evidence that participants believed the behaviour may have implications for working effectively, and would be influenced by the physical environment and work culture. A number of interventions to promote or modify salient beliefs have been suggested, and future research may consider the influence of these interventions on TPB determinants of, and actual reduced sitting behaviour. Such theory-informed interventions would provide important and useful insight into the psychological mediators of behaviour change. Further research would also be valuable to consider the generalisability of the findings of this belief elicitation study to non-U.K.-based office workers. Finally, whilst belief elicitation studies have value as standalone studies, future research could test the full TPB pathway from beliefs to behaviour to examine explanatory power of these variables for standing and light movement behaviour in the workplace. A full examination of the value of the TPB would build on previous research (Prapavessis et al., 2015), but with a focus on reducing sitting instead of current sedentary behaviour, and add to the growing literature using psychological theory to understand and enhance sedentary behaviour.
